# Keep Yourself Posted on Current Events

**Published:** 1897-09

**Authors:** 


### KEEP YOURSELF POSTED ON CURRENT EVENTS.

Twenty-eight horses have recently been destroyed in Indiana suffering from glanders, said to be from cheap horses brought in the State and sold to farmers. The State Live-stock Sanitary Commission are investigating the condition of horses in several localities.

Another one of the schemes recently adopted to give notoriety by a veterinary surgeon in a large city consists of a large, illuminated blotting-pad, with the picture of an ambulance and flying horses. What next?

In the Massachusetts volunteer militia regulations there is a clause referring to the duties of veterinary surgeons of each brigade, which reads as follows: "They will thoroughly inspect all horses reported for duty, and they are authorized to reject horses unfit for service and order that they shall not be returned for allowance on bills or pay-rolls. If possible, they will inspect the horses for artillery and cavalry prior to their leaving their home posts. If it is necessary, in order to accomplish this, to have the duty performed the day before camp, the veterinary surgeons will perform this duty on those days and be returned on pay-rolls for extra duty."

Lieutenant-colonel and Medical Director Clark, of the First Brigade, in his report (Public Document No. 7, 1897) to the Adjutant-General of the Massachusetts volunteer militia, speaks of the hospital facilities afforded by First Lieutenant and Veterinary Surgeon Osgood for the accommodation of sick and disabled horses used for military purposes. Surgeon-General Blood further recommends for the benefit of the service that Lieutenant F. H. Osgood, veterinary surgeon of the First Battalion and Artillery, and Lieutenant Austin Peters, First Cavalry, should be promoted to the rank of captain and attached to brigade headquarters.

Lincoln, Nebraska, is building a sheep-dipping vat capable of treating 10,000 a day. A triple swim a distance of ninety feet and a two-minute period in the dip, with very ample covering sheds to dry off in, are the complement of requirements. Sheep-owners will have their choice of any of the approved sheep-dips.

Yvette, the pet cocker-spaniel bitch of Mrs. Finlay Anderson, the novelist, is dead. Her owner will prepare for print a biography of the animal, that her memory may be better preserved among those who had not the privilege of her acquaintance.

Alaska will have established shortly within its borders an agricultural experiment station. The sum of fifteen thousand dollars has been appropriated for this under the Hatch law establishing these stations.

An important contribution at Nashville will be by Dr. Veranus A. Moore, of the Bureau of Animal Industry, on "A Cause of Death among Swill-fed Pigs, based upon Experiments." As this is a very important subject among the boards of health of all large cities, it is sure to awaken a deal of interest among all city inspectors.

Boards of health having veterinarians on their staff of officers should see that these members be sent to the annual conventions of the United States Veterinary Medical Association, where are considered so many vital questions relating to the field of their work.

Draught-horses in Chicago sold in August as high as $190 a piece. They are reported scarce, and orders are difficult to fill. An Ohio newspaper was shown the editor of the JOURNAL in August, quoting the sale of a heavy draught-horse for $146.50, which could have been bought by a dealer at less than $100 sixty days prior.

The *Weekly Picayune*, of New Orleans, of August 26th, gives a hearty welcome to the first meeting of the U. S. V. M. A. in the South. It goes farther in the education of the public of our mission to the South in a brief review of the work it is doing, its high standing among the learned professions, and reference to the excellent class of professional members of which it is composed. Surely with such greetings as this we must all feel a high sense of honor when numbered among its membership.

The department of civil service will conduct examinations on meat-inspection on October 25th at the following places: Montgomery, Alabama; Little Rock, Arkansas; San Francisco and Los Angeles, Cal.; Denver, Col.; Hartford, Conn.; District of Columbia; Jacksonville, Fla.; Atlanta, Ga.; Chicago and Peoria, Ills.; Indianapolis, Ind.; Des Moines, Iowa; Wichita, Kansas; Louisville, Ky.; New Orleans, La.; Portland, Maine; Boston, Mass.; Detroit and Grand Rapids, Mich.; Duluth and Minneapolis, Minn.;

Vicksburg, Miss.; St. Louis and Kansas City, Mo.; Helena, Mont.; Omaha, Neb.; Concord, N. H.; Albuquerque, N. M.; New York, Albany, and Rochester, N. Y.; Wilmington, N. C.; Cincinnati and Cleveland, Ohio; Oklahoma City, Oklahoma; Portland, Ore.; Pittsburg, Philadelphia, and Harrisburg, Pa.; Providence, R. I.; Charleston, S. C.; Sioux Falls, S. D.; Nashville and Knoxville, Tenn.; Fort Worth, San Antonio, and Houston, Texas; Salt Lake City, Utah; Burlington, Vt.; Richmond, Va.; Seattle, Wash.; Parkersburg, W. Va.; Milwaukee, Wis.; Cheyenne, Wyo.

Applicants should exercise the utmost care in specifying the date and place selected for examination, and should make no requests for changes in the schedule. All applications must be on file in the office of the commission on the proper blank forms at least ten days prior to the date selected for examination.

Dr. George Hopkins, of Cleveland, Ohio, reports successful results in tetanus with serum prepared from the blood of goats made immune to tetanus by successive inoculations of the tetanus bacilli.

Formalin is highly commended for the treatment of ringworm. As these are often very annoying in horses and cats, we hope some of our readers will give it a trial and report their experiences.

The use of Sanmetto in azoturia is very highly extolled by Veterinarian F. J. Bliss, of Earlville, Ills., who reports its use in over fifty cases in various stages of the disease with uniformily good results. Its prompt action in cleaning up the characteristic color and offensive odor and abatement of the alarming symptoms of the disease are especially noted, with marked relief to the animals.

At a meeting of the French Academy of Medicine, held in Paris on the 27th of July last, Dr. Nocard stated that the Pasteur laboratories in Paris had dispensed 7000 doses of 10 c.c. each of tetanus antitoxin, which had been used for the preventive treatment of 3100 animals located in districts where tetanus was endemic. Not a single death from tetanus occurred. One horse alone, treated five days after the infliction of the wound, had tetanus, but the disease was mild. During the same time the same veterinarians had observed 259 cases of tetanus in animals that had not been treated preventively with the antitoxin. Dr. Nocard states that there is no doubt of the utility of preventive injections of tetanus antitoxin in veterinary practice. The treatment of tetanus, when once thoroughly declared, is difficult and uncertain. Its prevention, therefore, is of prime importance.

In 1896 the United States exported 491,565 head of sheep, nearly four times the number exported in 1889. Exports of fresh beef, hams, and oleo have all increased in very great proportions.

Illinois has a lien law for registered stallions.

The Government inspection of stallions used for breeding purposes is still being agitated and gaining fresh adherents. The plan suggested that their inspection be carried on by State Veterinary Sanitary Boards or State Veterinary Associations under Government direction seems to meet with much favor.

Providence, Rhode Island, will have a horse improvement association, among many other leading attractions, to stimulate interest in horse-breeding and the ownership of handsome animals.

Two of Erie's leading veterinarians recently came to blows, followed by an arrest and prosecution, the outgrowth of some examinations made of cattle in that district for tuberculosis.

The Chicago Light Cavalry is the latest addition to mounted troops of the "Windy City." Surely the needs of horses are multiplying, and with the higher prices daily prevailing the signs of the times are more promising.

The Mississippi Experiment Station recommends the following application for the homfly: Two parts of fish-oil or cottonseed-oil and one part of pine-tar. Reapply with a brush every three to six days.

Tennessee and Arkansas cattle have been denied admittance to Illinois, on account of Texas fever, by proclamation of the Governor of Illinois.

A "Horseman's Protective Association," with principal office in New York, was incorporated last week at Albany. The objects are the fostering and protecting of the interests of horse-owners, trainers, and jockeys; the improvement of the breeds of throughbred horses through trials of speed and otherwise; the settling of differences between members, and the promotion of more enlarged and friendly intercourse among all classes engaged in owning, training, racing, or riding thoroughbred horses.

Dr. N. D. Daddy, of Loett, Indiana, is quoted as having proven conclusively that for driving horses clover hay is as satisfactory for an exclusive diet as it is for work-horses.

A practical device invented by Mons. Zimmerman, of Paris, France, for the prevention of runaways, has already become a familiar sight on the boulevards of that city. The apparatus is a woven-wire muzzle which extends over the horse's face, just covering the nostrils, and is connected with the reins by stout straps. When the horse becomes excited and attempts to run away the reins are pulled tight, thus causing the muzzle to close over the nostrils and mouth, producing immediate suffocation. A number of vicious horses were experimented upon with the device, and in every instance the animal was almost instantaneously stopped. The only real objection to the apparatus is its unsightliness.—*Spirit of the Times.*

A duty has been imposed on Canadian horses unloaded at Skaguay, Alaska, and it is feared that this action will cause discontent among American miners; but the duty was paid, although under protest. The collector also assumed the duty of appraising the horses according to their value in Alaska.

The *Country Gentleman* quotes Prof. Cottrell, of the Kansas State Agricultural College, referring to the recent reports of tuberculosis made through the public press. It can be tracked back at least to 1880, since which time it has been gradually spreading, though the general health of the herd continued excellent for a dozen years after that date. Tuberculin-tests were begun in February, 1894; but the whole herd was not gone through till last winter, when eighteen out of fifty-two were found affected, and were isolated. A second and more searching investigation is to be made on October 20th. Meanwhile no animals will be sold, none purchased, and no milk used for dairy purposes or for feeding animals.

A million and a half pounds of canned beef was recently prepared and shipped by a Kansas City firm for use in the Japanese army.

The United States is said to have had over forty million hogs at the beginning of the year, Iowa alone having nearly four million.

At last the two-minute record has been broken, and Star Pointer stands to-day king of trotters the world over. 1.59¼ points the beacon-mark of attainment in the breeding of trotters, and the wonderful strides to this great feat make its attainment all the more to be proud of. Our good friend, Dr. Bailey, of Maine, still lives, and though he had not expected to see this accomplished in his lifetime, we trust that many more years may be his to enjoy, and that the breaking of this record may yet be reached.

Over fourteen million horses within our borders on January 1, 1897. Iowa with over one million, Texas and Illinois a like number, while Rhode Island has but 10,129, with an average valuation of $75.25 each; Kentucky with 400,879, valued at over $13,000,000, or $32.57 per head, while those of Oklahoma are appraised at $13.40 per head. Texas leads in mules, with 261,428 in number to her credit, while Georgia has the greatest value invested, her 164,380 averaging $62.95 each. New Jersey averages the value of her mules at $76.70, while New Mexico only places $19.15 as the average value. The mules in the entire country are estimated at 2,213,654 and valued at $92,302,000, or an average of $41.65. From 1892 to 1897 the value of horses decreased nearly $600,000,000, while the number fell off only one million.

Another pair of draught-horses recently brought the sum of $465 at one of the Chicago auction-marts.

A freak of nature has been recently discovered in Kentucky. It is a pony forty inches high and five feet five inches in length. Its head is six inches longer than its neck, and it has a breast like a cow and hips like a hog. It possesses twenty-eight ribs and four stifle-joints, and is double-jointed in all joints. It is four years old and of Norman breed, and weighs 285 pounds. It is owned by Mr. Mason Gordley.

